# Exploring a Role for the Arabidopsis TIR-X Gene (TIRP) in the Defense Against Pathogenic Fungi or Insect Herbivory Attack

**DOI:** 10.3390/ijms26062764

**Published:** 2025-03-19

**Authors:** Shraddha Neufeld, Michael Reichelt, Sandra S. Scholz, Przemysław Wojtaszek, Axel Mithöfer

**Affiliations:** 1 Research Group Plant Defense Physiology, Max-Planck Institute for Chemical Ecology, 07745 Jena, Germany; swadurkar@ice.mpg.de; 2 Department of Molecular and Cellular Biology, Adam Mickiewicz University, 61-712 Poznan, Poland; przemow@amu.edu.pl; 3 Department of Biochemistry, Max-Planck Institute for Chemical Ecology, 07745 Jena, Germany; reichelt@ice.mpg.de; 4 Department of Plant Physiology, Matthias Schleiden Institute of Genetics, Bioinformatics and Molecular Botany, Friedrich-Schiller-University, 07743 Jena, Germany; sandra.scholz.da@gmx.de

**Keywords:** *Arabidopsis*, plant immunity, phytohormones, TIR-X, *Verticillium dahliae*, *Alternaria brassicicola*, *Spodoptera littoralis*

## Abstract

Plants are challenged regularly with multiple types of biotic stress factors, such as pathogens or insect herbivores, in their environment. To detect and defend against pathogens, plants have evolved an innate immune system in which intracellular receptors in the so-called effector-triggered immunity play a vital role. In *Arabidopsis thaliana* the Toll/interleukin-1 receptors (TIRs) domain is related to intracellular immunity receptors, for example in TIR-NBS-LRR (TNL) proteins. Among the TIR domain carrying proteins, very little is known about the function of the TIR-X proteins. Here, we focus on the recently described TIR-X (TIRP; At5g44900) to analyze its role in phytohormone-mediated plant defense through gene expression and phytohormone quantification. Therefore, we employed two fungal pathogens, the necrotrophic *Alternaria brassicicola* and the hemibiotrophic *Verticillium dahliae*, to infect *A. thaliana* WT (Col-0), TIRP knock-out, and TIRP overexpressing lines for comparative analyses. Furthermore, we included the insect herbivore *Spodoptera littoralis* and a treatment with *S. littoralis* egg extract on the plants to analyze any role of TIRP during these attacks. We found that both *A. brassicicola* and *V. dahliae* infections increased TIRP gene expression systemically. The salicylic acid content was higher in the TIRP overexpressing line, corresponding to a better *S. littoralis* larval growth performance in feeding assays. However, since we never observed clear infection-related differences in jasmonate or salicylic acid levels between the wild type and the two transgenic *Arabidopsis* lines, our results rule out the possibility that TIRP acts via the regulation of phytohormone synthesis and accumulation.

## 1. Introduction

Plants are exposed to multiple biotic and abiotic factors in the environment. To protect themselves from pathogens, such as bacteria, fungi, and viruses, plants have evolved innate immunity [1]. There are two layers of the plant immune system, pattern-triggered immunity (PTI) and effector-triggered immunity (ETI). Both are initiated by the physical interaction between pathogen-derived signaling compounds and their corresponding receptors on the plant’s side. The first layer employs the transmembrane pattern recognition receptors (PRRs) of the plasma membrane, which recognize microbial- or pathogen-associated molecular patterns (MAMPs or PAMPs). The second layer performs inside the cell and interacts with the effectors provided by the pathogens. Here, disease resistance proteins encoded by (R) genes [2] are involved. The major resistance (R) protein class is composed of a nucleotide-binding site (NBS) domain and a C-terminal Leucine Rich Repeat (LRR) domain [3]. The N-terminus of the NBS-LRR resistance proteins either carries a Toll/Interleukin-1 receptors (TIRs) homology domain or a coiled-coil (CC) domain. This defines two further types, one along with an N-terminus localized TIR is known as TIR-NBS-LRR (TNL) and the second with an N-terminal coiled-coil (CC) is called CC-NBS-LRR (CNL) [4]. Within these TIR-NBS-LRR proteins, there are two protein families containing TIRs. One consists of TIRs and a NBS (TN) but lacks LRR; the other class known as TIR-X (TX) lacks both NBS and LRR domains [4]. The absence of both LRR and NBS domains in TX distinguishes it from other plant R gene-encoded proteins [5]. The role of the ‘X’ domain in TIR-X is unclear; they are either called atypical or undefined domains [5,6]. According to Meyers et al. [5], there are 30 TIR-X genes in *Arabidopsis*. Very little is known about TIR-X proteins in plants. Few TIR-X proteins have a transmembrane domain (TMD) [7]. It is a feature that most of the TIR family lacks [8]. For example, the amino acid sequence analysis of a TIR-X protein (named TIRP, At5g44900) predicted a single TMD by using TMHMM server version 2.0 [8]. The presence of the TMD in TIRP makes it different from TIR-NBS-LRR and many other TIR-X proteins [7,8]. A soluble protein, TX14 (At2G32140), is the closest TIRP homolog which is supposed to be involved in salicylic acid signaling and defense responses [9]. Another study shows that the *Agrobacterium tumefaciens*-meditated transient overexpression of the *Arabidopsis* TIR-X protein AtTX21 in *Nicotiana benthamiana* results in a hypersensitivity response (HR) and higher resistance against the bacterium *Pseudomonas syringae* pv tomato DC3000 and the fungus *Fusarium oxysporum* [10]. Additionally, a TIR-X (At2g32140) overexpressing line showed a strong dwarf phenotype when plants grew at 28 °C rather than at 22 °C, as well as an increase in defense-related gene expression [9]. In our previous study, TIRP was found to interact with the plant γ-secretase, a multi-subunit protease complex that is known to have an important role in cellular trafficking and apoptosis [8]. Furthermore, a transient overexpression of TIRP in *N. benthamiana* showed necrosis in leaves, suggesting a HR [8]. In addition to these studies, very little is known about the function and role of TIR-X in plant immunity.

In plant immunity, the pivotal role of phytohormones in the regulation of the plant’s defense signaling network is known. In addition, plant hormones promote growth in plants, play an important role in development and metabolism, and are signals to protect from different environmental stresses [11]. Plant pathogens have evolved to facilitate infection by producing toxins and effectors that harm or manipulate hormonal crosstalk [12]. Evidence is available showing that pathogens try to demolish hormonal pathways not only to lower plant immunity but also to accelerate pathogen dissemination and enhance nutrient acquisition [12]. Many types of harmful pathogens are present in the environment among which fungi are a dominant causal agent of plant diseases [13]. Fungal pathogens are categorized into biotrophic, hemibiotrophic, and necrotrophic depending upon their interaction with the host plant [14]. Likewise, another major threat to the plant is herbivory attack. Herbivory can negatively affect a plant’s survival, immunity, and reproductive output, which can influence plant population dynamics [15]. To survive and nurture the next generation of these herbivores, they lay eggs on plants which is a preparation technique for the next plant attack via infestation. In this study, we address the question of a putative function for TIPR when the plant is under attack from a pathogenic fungus or an herbivorous insect. Therefore, we employed two types of fungal pathogens, the hemibiotrophic *Verticillium dahliae* and the necrotrophic *Alternaria brassicicola*, and an herbivore attack by the larvae of *Spodoptera littoralis* and analyzed the phytohormones as markers of induced defense responses in the plants. TIRP knock-out and overexpressing lines were used to gain insights into a hypothetical role for TIRP in plant immunity.

## 2. Results

### 2.1. Fungal Treatments

**Figure 1:**
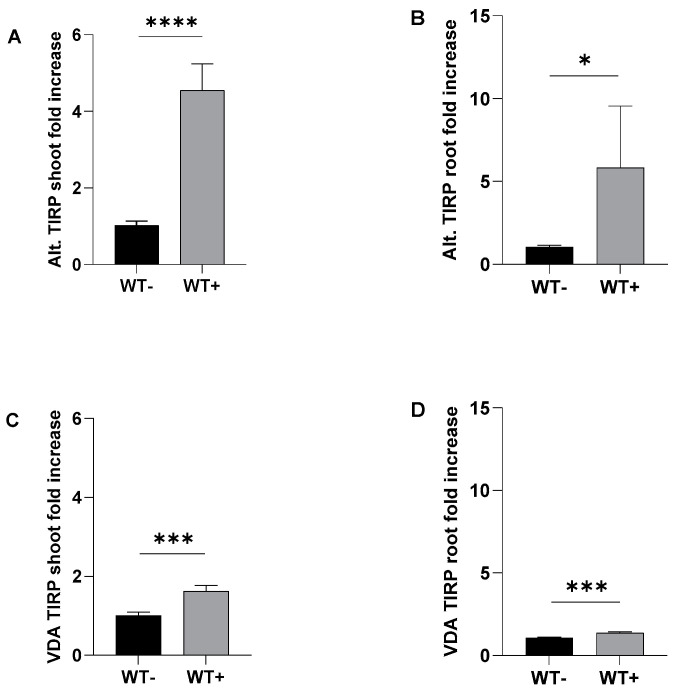
The TIRP gene expression levels in *Arabidopsis* wild type (WT) after *Alternaria brassicicola* (Alt) or *Verticillium dahliae* (VDA) fungal infections. Seedlings were infected at the age of 10 days. *A. brassicicola* was applied to a single leaf of each plant while *V. dahliae* was applied to the roots. After 7 days of infection, the shoot and root were separated, harvested, frozen, and used for RTqPCR analysis. The figure shows the TIRP levels (±SE) detected in (**A**) shoots and (**B**) roots upon *A. brassicicola* treatment and in (**C**) shoots and (**D**) roots upon *V. dahliae* treatment. The statistical differences were tested with Student *t*-test using GraphPad Prism and considered significant when *p* ≤ 0.05. (*) represents *p* ≤ 0.05, (***) represents *p* ≤ 0.001, and (****) represents *p* ≤ 0.0001. *A. brassicicola* treatment WT − n = 96, WT + n = 80 and *V. dahliae* treatment WT − n = 112, WT + n = 104, both from four independent replications. The label (+) means with fungal infection; (−) means without fungal infection.

In order to find out whether or not TIRP is involved in the defense reactions against fungal infections, first the TIRP mRNA levels in *Arabidopsis* wild type (WT) were analyzed by RTqPCR during the fungal infection with necrotrophic *A. brassicicola* or hemibiotrophic *V. dahliae* (Figure 1). We found that the TIRP gene expression in *A. brassicicola*-infected *Arabidopsis* leaves and roots was significantly upregulated compared to non-infected controls (Figure 1A,B). The upregulation of TIRP in roots shows that the *A. brassicicola* infection caused a systemic response (Figure 1B). In the case of the *V. dahliae* infection, the TIRP expression was less but was significantly upregulated in both shoots and roots (Figure 1C,D).

**Figure 2:**
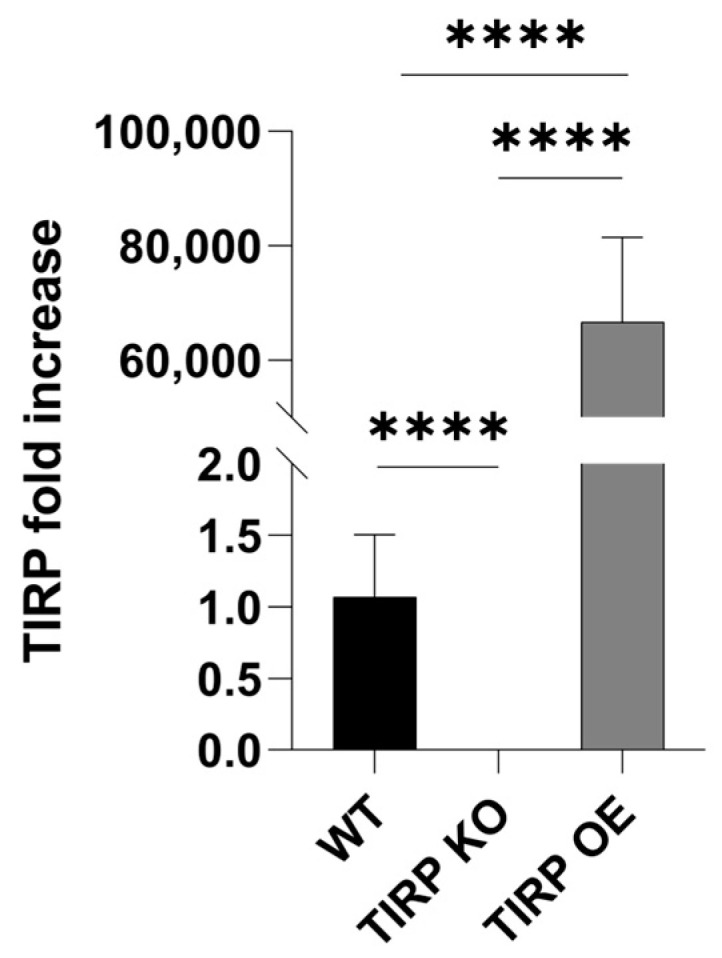
IRP gene expression in 5-week-old *Arabidopsis* wild type (WT), TIRP knock-out (TIRP KO), and TIRP overexpressing (TIRP OE) lines without treatment. Statistical differences were analyzed by using Student *t*-test comparing pairs. Statistical differences were considered significant when *p* ≤ 0.05. (****) represents *p* ≤ 0.0001. WT n = 12, TIRP KO n = 18, and TIRP OE n = 18.

**Figure 3:**
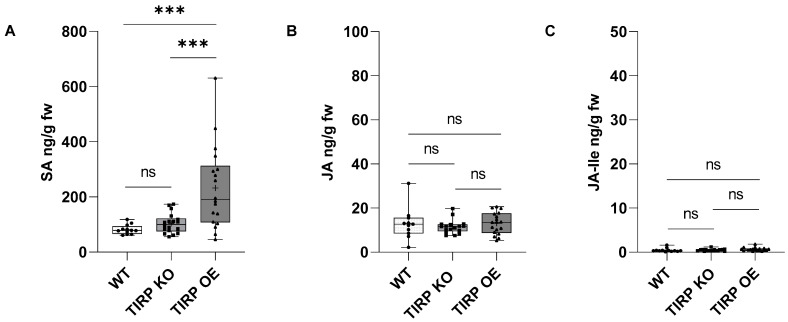
Jasmonate and salicylic acid levels in Arabidopsis WT, TIRP KO, and TIRP OE lines. Phytohormone levels (mean ± SE) were measured in 3-week-old plants without any treatment to plants. (**A**) Salicylic acid (SA), (**B**) Jasmonic acid (JA), and (**C**) Jasmonoyl isoleucine (JA Ile). Boxplots are shown (with center line, median; box limits, upper and lower quartiles; whiskers, 1.5× interquartile range). Statistical differences were calculated using two-way ANOVA and considered significant when *p* ≤ 0.05 (Tukey); ns = non-significant (*p* > 0.05), (***) represents *p* ≤ 0.001. WT n = 12, TIRP KO n = 18, and TIRP OE n = 18 from four independent replicates.

For further studies, we employed the TIPR knock-out (KO) and TIRP over-expressor (OE) lines. The TIRP levels without any treatment were analyzed in all three *Arabidopsis* lines using RTqPCR (Figure 2). The TIRP mRNA level in the TIRP KO line was undetectable, while in the TIRP OE line the level was significantly higher compared to the WT control (Figure 2). In all lines, the infection with *A. brassicicola* or *V. dahliae* was successful (Figure S1) and strongly affected the fresh weight of the shoots and roots (Figure S2). Moreover, we analyzed defense-related phytohormones [16] in these lines to determine if there are any differences in the levels of SA, JA, and JA-Ile (Figure 3). In contrast to the two jasmonates, JA and JA-Ile, which showed no differences in their amounts between the three *Arabidopsis* lines, the SA level in the TIRP OE line was significantly higher compared with WT and TIRP KO.

**Figure 4:**
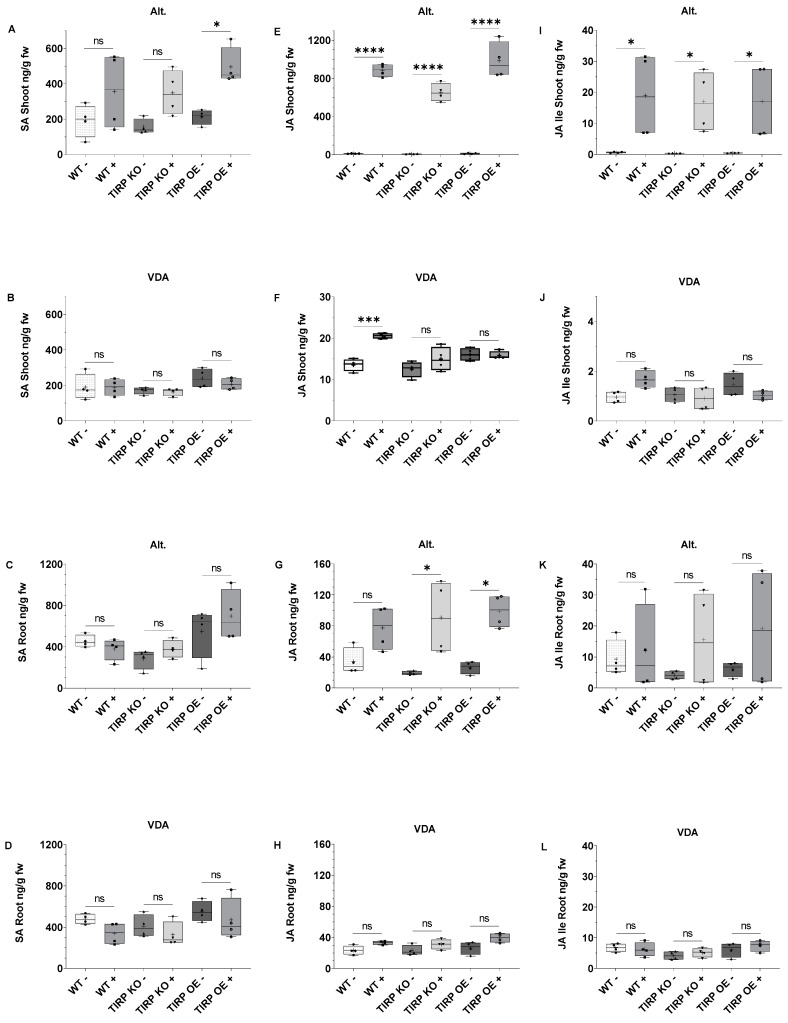
Phytohormone (SA, JA, and JA-Ile) levels in shoots and roots in fungal-infected Arabidopsis lines. Ten-day-old seedlings of Arabidopsis WT, TIRP KO, and TIRP OE lines were infected with *A. brassicicola* (Alt) (**A**,**C**,**E**,**G**,**I**,**K**) on leaf or with *V. dahliae* (VDA) (**B**,**D**,**F**,**H**,**J**,**L**) on roots. After 7 days of infection, all plant shoots and roots were separated, harvested, and analyzed for phytohormone contents before and after treatment. (**A**) SA in shoots (*A. brassicicola*), (**B**) SA in shoots (*V. dahliae*), (**C**) SA in roots (*A. brassicicola*), (**D**) SA in roots (*V. dahliae*), (**E**) JA in shoots (*A. brassicicola*), (**F**) JA in shoots (*V. dahliae*), (**G**) JA in roots (*A. brassicicola*), (**H**) JA in roots (*V. dahliae*), (**I**) JA-Ile in shoots (*A. brassicicola*), (**J**) JA-Ile in shoots (*V. dahliae*), (**K**) JA-Ile in roots (*A. brassicicola*), and (**L**) JA-Ile in roots (*V. dahliae*). Boxplots are shown (with center line, median; box limits, upper and lower quartiles; whiskers, 1.5× interquartile range). Statistical differences were calculated using two-way ANOVA and considered significant when *p* ≤ 0.05 (Tukey); ns: non-significant (*p* < 0.05), (*) represents *p* ≤ 0.05, (***) represents *p* ≤ 0.001, and (****) represents *p* ≤ 0.0001. *A. brassicicola* treatment WT − n = 96, WT + n = 80, TIRP KO − n = 84, TIRP KO + n = 92, TIRP OE − n = 88, and TIRP OE + n = 84. *V. dahliae* treatment WT − n = 112, WT + n = 104, TIRP KO − n = 112, TIRP KO + n = 108, TIRP OE − n = 108, and TIRP OE + n = 112 from four independent replications.

Next, the shoot and root samples from the *A. brassicicola* or *V. dahliae* infection experiments were checked for phytohormone levels (Figure 4). When *A. brassicicola* was applied to the leaves of the seedlings, all three tested *Arabidopsis* lines showed a pattern of elevated SA levels (Figure 4A). However, only in the TIRP OE line was the SA level significantly higher upon infection when compared to the non-treated control. In case of the *V. dahliae* infection, the shoots did not show any significant changes in SA contents (Figure 4B). In the roots of all three *Arabidopsis* lines, the SA levels did not change at all, neither upon *A. brassicicola* or on *V. dahliae* infection (Figure 4C,D). The differences in the JA levels in the shoots of the *A. brassicicola*-treated leaves of all three *Arabidopsis* lines were found to be significantly higher compared to untreated controls (Figure 4E). The same result was found for JA-Ile (Figure 4I). In the *V. dahliae* treatment the JA level in the shoots of the WT was significantly higher, which was not the case with TIRP KO and TIRP OE (Figure 4F). The JA-Ile level in the shoots of all the lines was not significantly changed with *V. dahliae* infection (Figure 4J). In the roots of TIRP KO and TIRP OE, the JA level was significantly higher with *A. brassicicola* infection than the WT (Figure 4G). The JA-Ile level did not change significantly in all three lines (Figure 4K). The *V. dahliae* infection showed no significant differences in the JA and JA-Ile levels in the roots of all the lines (Figure 4H,L).

### 2.2. Insect Herbivore Treatments

**Figure 5:**
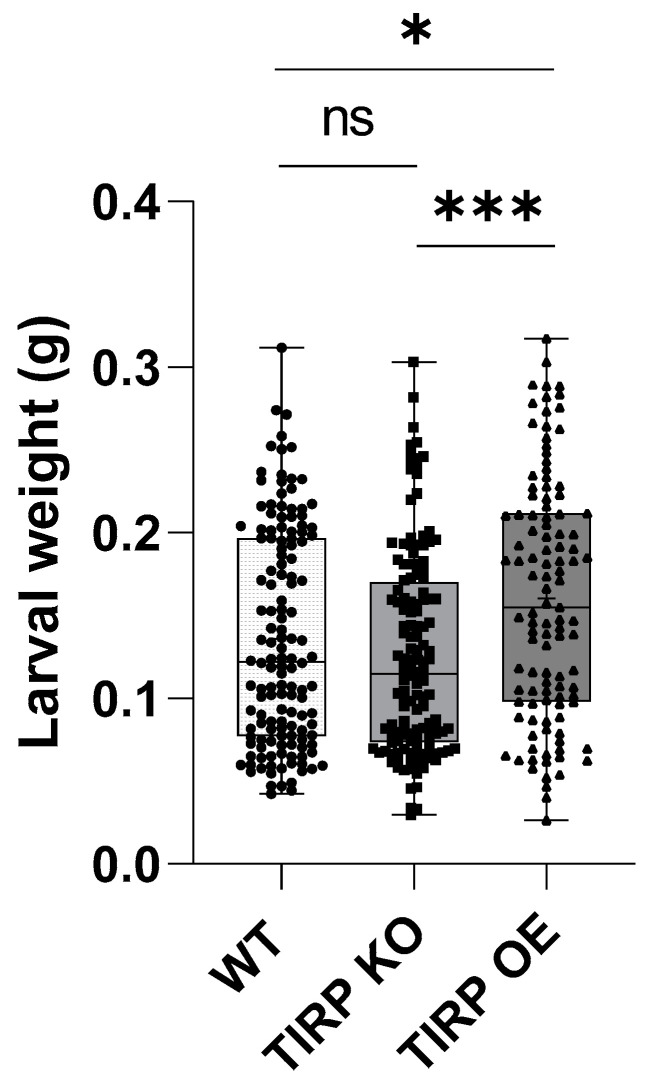
Spodoptera littoralis larval weight gain upon feeding on Arabidopsis WT, TIRP KO, and TIRP OE lines. First-instar *S. littoralis* larvae were left on 5-week-old plants for feeding. After 7 days larvae were removed and their weight was measured individually. Statistical differences were calculated using ordinary one-way ANOVA and considered significant when *p* ≤ 0.05 (Tukey). Boxplots are shown (with center line, median; box limits, upper and lower quartiles; whiskers, 1.5× interquartile range); ns: non-significant (*p* > 0.05); (*): *p* ≤ 0.05; and (***): *p* ≤ 0.001. WT: n = 141, TIRP KO: n = 140, and TIRP OE: n = 111, from five independent replicates.

Because TIRP might be involved in the defense response against herbivores, we included feeding experiments in the present study. Therefore, the larvae of *Spodoptera littoralis*, a generalist insect herbivore, were fed on WT, TIRP KO, or TIRP OE leaves. After one week of feeding their weight was measured (Figure 5). It was found that the larvae gained a significantly higher weight when they were feeding on the TIRP OE line compared with the WT control. This effect was more pronounced when the TIRP OE line was compared with TIRP KO lines (Figure 5).

**Figure 6:**
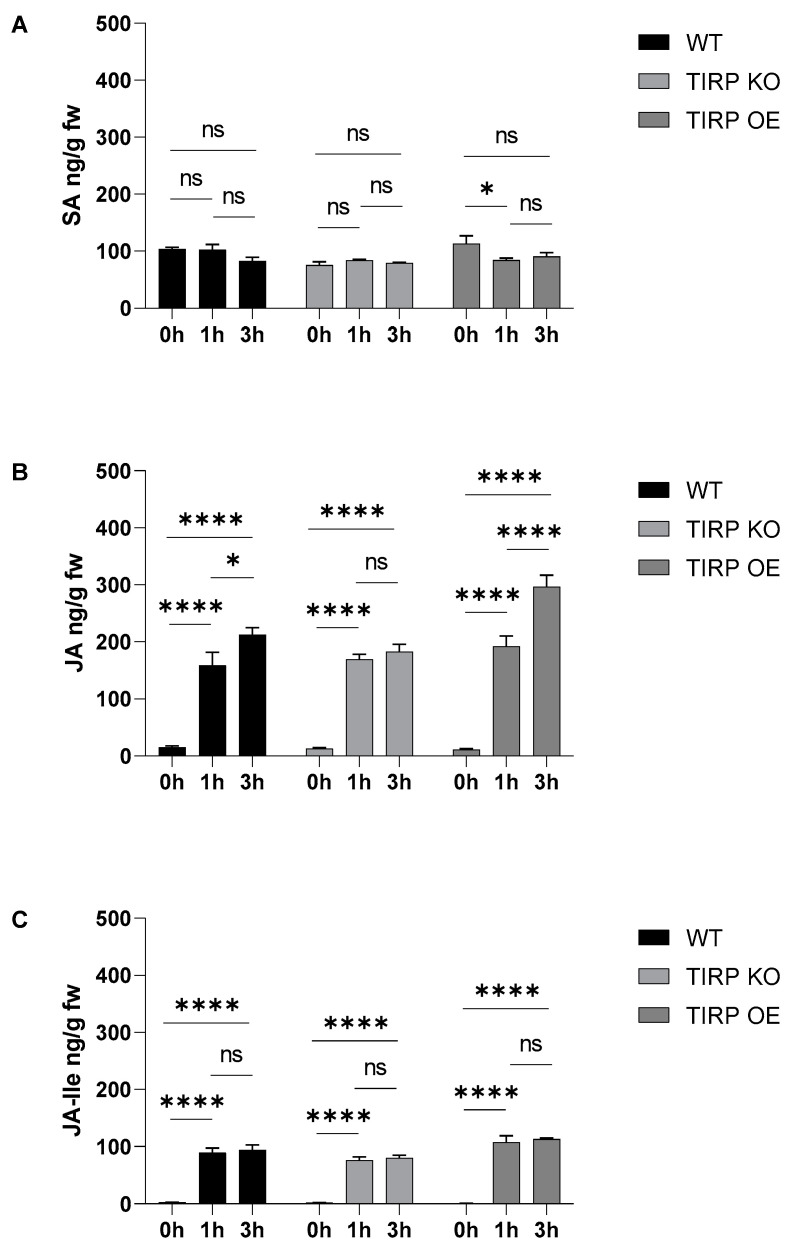
Phytohormone analysis of 5-week-old Arabidopsis WT, TIRP KO, and TIRP OE lines after third-instar *S. littoralis* larvae feeding. Feeding was allowed for 1 and 3 h then leaves were harvested for analysis. Levels (mean ± SE) of (**A**) Salicylic acid (SA), (**B**) Jasmonic acid (JA), and (**C**) Jasmonoyl isoleucine (JA Ile) are shown. Statistical differences were calculated using two-way ANOVA and considered significant when *p* ≤ 0.05 (Tukey); ns: non-significant (*p* < 0.05), (*) represents *p* < 0.05, and (****) represents *p* ≤ 0.0001, n = 10.

**Figure 7:**
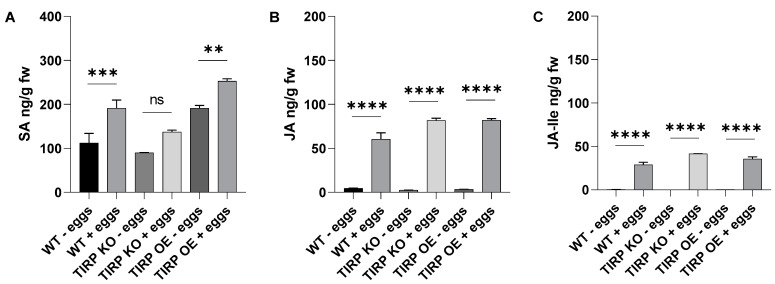
Phytohormone analysis of 5-week-old Arabidopsis WT, TIRP KO, and TIRP OE lines after treatment with *S. littoralis* egg extract. Eggs (approx. 100) were crushed and extract was applied on each leaf of 5-week-old plants. One single leaf per plant was treated with egg extract for 48 h and then harvested for analysis. Levels (mean ± SE) of (**A**) Salicylic acid (SA), (**B**) Jasmonic acid (JA), and (**C**) Jasmonoyl isoleucine (JA Ile) are shown. Statistical differences were calculated using ordinary one-way ANOVA and considered significant when *p* ≤ 0.05 (Tukey); ns: non-significant (*p* > 0.05), (**) represents *p* ≤ 0.01, (***) represents *p* ≤ 0.001, and (****) represents *p* ≤ 0.0001, n = 10.

To investigate the effects of short feeding times on the phytohormones, *S. littoralis* larvae were fed for 0 h, 1 h, and 3 h on WT, TIRP KO, and TIRP OE plants (Figure 6). SA in WT and TIRP KO for 0 h, 1 h, and 3 h was found at the same levels with no significant difference. Only a small difference was detected in TIRP OE between 0 and 1 h (Figure 6A). In contrast, compared to the controls, in all the *Arabidopsis* lines there was a highly significant increase in JA and JA-Ile due to *S. littoralis* feeding after only 1 h of treatment. The JA content further increased at 3 h (Figure 6B,C). The oviposition of a butterfly species, *Pieris brassicae*, as well as the application of the *P. brassicae* egg extract on *A. thaliana* leaves caused an increase in the SA level [17]. Therefore, in the present study, phytohormone analysis was performed upon plants treated with *S. littoralis* egg extract. Here we found that in all *Arabidopsis* lines the SA, JA, and JA-Ile levels increased significantly (Figure 7).

## 3. Discussion

### 3.1. Alternaria brassicicola and Verticillium dahliae Treatments

Plants face multiple challenges caused by pathogenic microbes or herbivores. After detecting the attacker by various receptors, plants activate different layers of defense responses through an organized signaling network. Among others, plant TIR domain proteins are involved in innate immunity. However, the experimental data available on the involvement of the TIR gene in the immunity against necrotrophic and hemibiotrophic fungi or insect herbivory are limited. In a previous study, a gene resistance to Leptosphaeria maculans 3 (RLM3) was found to encode a putative TIR-NB class protein providing resistance against the hemibiotrophic fungus *Leptosphaeria maculans*, the necrotrophic pathogen *Botrytis cinerea,* and necrotrophic fungi, like *A. brassicicola* and *A. brassicae* [18,19]. Moreover, when the transgenic TX and TN (*At*TN10, *At*TN11, *At*TN21, *At*TX21) overexpressing lines were infected with the hemibiotrophic fungus *F. oxysporum*, an increased resistance to the pathogens was found [10]. In addition, upon the external application of the phytohormone SA on the leaves of these overexpressing lines a subset of the TX and TN genes was found to be induced [10]. Up to now, TX in general has not been studied much. Therefore, our study was conducted to gain more insight into a possible role of TX, especially TIRP, in plant immunity and the possible links to phytohormone-regulated processes.

We found that in *Arabidopsis* WT the infection with the necrotrophic *A. brassicicola* caused an induction of the TIRP gene in both shoots and roots. Although to a lesser extent, the same holds true for infection with *V. dahliae* (Figure 1), suggesting that the pathogen attack influences TIRP gene expression. It has been reported that many effectors of hemibiotrophic pathogens can suppress the host plant’s LRR-RLP-mediated resistance to pathogens. For example, in a hemibiotrophic oomycete (*Phytophthora infestans*)-infected tomato plant, the pathogen’s secretion of SNE-1 (suppressor of necrosis-1) can inhibit NLP (Nep1-like proteins)-induced cell death [20]. The TIR-X group lacks both the LRR and NBS regions [5]. Thus, it might be possible that such a suppression effect caused the attenuated induction of TIRP in the *Arabidopsis* WT upon *V. dahliae* treatment.

It is well known that the phytohormones SA and JA play a major role in plant immunity [21]. SA-mediated defenses protect plants from biotrophic and hemibiotrophic pathogens, while JA protects plants from necrotrophic pathogens or herbivores [22,23,24]. Thus, we further analyzed these phytohormones. In non-infected plants, a slight but significantly higher constitutive level of SA was detected only in the TIRP OE line (Figure 3). Upon *V. dahliae* infection, in all *Arabidopsis* lines almost no jasmonate or SA induction was found, neither in shoots nor in roots (Figure 4), although infection was successful (Figures S1 and S2). This confirms the results from another study where jasmonates and SA were detected later in the *V. dahliae* infection phase, after 21 days [25]. This was completely different upon *A. brassicicola* infection. Here, jasmonates were induced both in shoots and roots and, in addition, SA was also found in shoots (Figure 4). Actually, in *Arabidopsis* SA and JA are seen to have an antagonistic nature [26]. However, NPR3 and NPR4, which are receptors of SA, have been shown to function opposite to NPR1, another proposed SA receptor, further activating JA signaling during effector-triggered immunity and resulting in the induction of both SA and JA [27,28]. Strikingly, there were no obvious differences observed between the WT, transgenic, and knock-out lines in our study. This suggest that TIRP does not have a clear role in the phytohormone-mediated defense responses after fungal pathogen attack. This conclusion is supported by the results shown in Figures S1 and S2, indicating no differences in the level of infection and the plant’s weight loss upon infection with either fungus. However, as we performed the experiments with seedlings, we cannot exclude the possibility that in plants with different developmental stages the situation could be slightly different.

### 3.2. Spodoptera littoralis Treatments

Upon an herbivore attack on plants, jasmonates are induced as an immune response [29,30]. To the best of our knowledge, there are no studies on any role of TIR in the defense against herbivory. Just one transcriptome study in chickpeas (*Cicer arietinum*) found that upon artificial wounding the TIR gene expressions were not affected or even reduced [31]. Addressing this open question, we found no *TIRP* induction in the *Arabidopsis* WT upon feeding up to 3 h. In a feeding assay where the *Spodoptera littoralis* larvae were allowed to feed on *Arabidopsis* WT, TIRP KO, or TIRP OE lines for 7 days, the larvae that grew on TIRP OE plants gained slightly more weight compared to the other lines (Figure 5). As there was no naturally occurring higher jasmonate level in this line (Figure 3), induced defense should be the reason behind this result. Indeed, the short-time *S. littoralis* feeding experiment showed a fast accumulation of both jasmonates within 1 h in all lines, with the highest enhancement of JA after 3 h in TIRP OE (Figure 6). However, again these results indicate that the TX protein under investigation, TIRP, is obviously not involved in the defense against herbivore feeding.

Another plant–insect interaction that causes defense is due to oviposition. Previously, studies dealing with *Pieris brassicae* oviposition on *Arabidopsis* have shown that SA accumulates at the site of oviposition causing programmed cell death at the site [17,32]. Moreover, herbivory-responsive genes were reported to be compromised, which resulted in increased attacks by chewing insects [17]. They also found that a generalist herbivore (*S. littoralis*) grew better on plants. Similar results were found upon treatment with an egg’s extract [17]. In one of these studies egg deposition was reported to induce lipase-related proteins (EDS1, PAD4, and SAG101), which play a role in transducing redox signals upon biotic and abiotic stresses [32]. Interestingly, TNL (TIR-NBS-LRR) proteins are known to recruit EDS1, PAD4, and SAG101 proteins to signal pathogen recognition and trigger hypersensitive (HR) plant cell death [33]. Recently in a cabbage (*Brassica nigra*) plant, the oviposition by cabbage white butterflies (*Pieris* spp.) triggered HR-induced cell death to reduce the egg’s survival [34]. The further genetic mapping of those HR cell death leaf samples identified a single locus responsible for cell death named PEK (Pieris egg-killing) [34]. This PEK locus was reported to encode intracellular TNL receptor proteins [34]. When the *S. littoralis* egg extract was applied to the leaves of *Arabidopsis* WT, TIRP KO, or TIRP OE for 48 h a significantly higher increase in SA in the TIRP OE line was observed compared to the others (Figure 7A). This supports the hypothesis that SA accumulates due to the egg’s components or elicitors, together with jasmonates that increase as well (Figure 7B,C). Thus, we cannot exclude the involvement of TIR-domain-containing proteins in the defense of *Arabidopsis* upon oviposition but must exclude a role for TIRP.

## 4. Materials and Methods

### 4.1. Plant Material and Growth Condition

The *Arabidopsis thaliana* ecotype Columbia 0 (Col-0, wild type) seeds were obtained from the Department of Molecular and Cellular Biology, Adam Mickiewicz University, Poland. The T-DNA insertion TIRP knock-out line SALK 014983 (TIRP KO) was ordered from Nottingham Arabidopsis Stock Centre (NASC). The genotyping primers for the knock-out line were designed from the SIGnAL website (http://signal.salk.edu/cgi-bin/tdnaexpress, accessed on 14 January 2020). The primers can be found in Table S1. The transgenic TIRP overexpressed (TIRP OE) line was created by transforming *A. thaliana* Col-0 with the *Agrobacterium tumefaciens* floral dip method [35]. The binary vector pEarleygate103 [36] with a desired genetic fragment and driven by CaMV 35S promoter was used for transforming *A. tumefaciens* bacteria. The transformed *A. thaliana* was selected by using a BASTA treatment. The transgenic line used for the experiments was stable until at least the third generation. Further experiments after this step, except the fungal infections, were performed at the Max Planck Institute of Chemical Ecology, Jena, Germany. Therefore, after stratification of seeds at 4 °C for two days, the pots were transferred to a growth chamber and plants were grown with a photoperiod that consisted of 10 h dark/14 h light with a light intensity of 100 µmol m^−2^ s^−1^ along with 21 °C temperature and 50–60% humidity. The fungal experiments were performed at Friedrich Schiller University, Jena, Germany. Therefore, seeds of *Arabidopsis* wild type, TIRP KO, and TIRP OE were surface sterilized and grown on MS nutrient media on a Petri dish. The Petri dishes were sealed with a parafilm. After stratification for 48 h at 4 °C the seeds were transferred to a growth chamber at 22–24 °C with a 16 h light/8 h dark photoperiod, with 80 µmol m^−2^ s^−1^ light intensity for 8 days. The 8-day-old seedlings were transferred on PNM media Petri dishes lined with nylon membrane discs prior. Once plants were 10 days old, they were ready to infect with fungal spores.

### 4.2. Alternaria brassicicola Growth Condition and Co-Cultivation

*Alternaria brassicicola* (FSU-218) was received from Jena Microbial Resource Center, Jena, Germany. A fungal lawn was made by placing a 5 mm diameter of *A. brassicicola* plug as an inoculum on the center of a Petri dish of potato dextrose agar (PDA), which was made using a potato dextrose broth (PDB, 3.9 g 100 mL^−1^) with 1% Kobe agar, pH 5.6. The *A. brassicicola* culture was incubated at 22–24 °C in a growth chamber for two weeks under 12 h light/12 h dark illumination with 75% relative humidity. The *A. brassicicola* spores from PDA plates were collected by pouring 0.01% sterile Tween-20 solution and gently scraping with a spatula without disturbing the agar. The collected spores were filtered through 3–4 layers of sterile nylon membranes (pore size 75 µm). The spore solution was centrifuged, washed with 0.01% Tween-20, and repeatedly washed 3 times. The spores were diluted with 0.01% Tween-20. The spore count was determined by a Haemocytometer and was adjusted to 1 × 10^6^ per mL by diluting with 0.01% Tween-20. Once the spore solution was set to 1 × 10^6^ per mL it was ready to infect the plant’s leaf. From this spore solution, 2 µL was pipetted out and was placed as a droplet over a single leaf per plant. For the control group, 2 µL of sterile water on a leaf was used instead. The Petri dishes were kept in a growth chamber with 16 h light/8 h dark photoperiod and 80 µmol m^−2^ s^−1^ light intensity at 22–24 °C and sealed with a 3M^TM^ Micropore tape (3M Germany GmbH, Neuss, Germany). After 7 days of infection, the shoot and root of plants were cut, washed with sterile water, and wiped with tissue paper. Although the *A. brassicicola* spores were applied on a single leaf per plant, at the time of harvest all the adjacent leaves along with the infected leaf were collected together. The shoots and roots were collected separately in sterile Sarstedt Screw Cap Microtubes (Nümbrecht, Germany) with two sterile metal beads (4.5 mm diameter, Askubal, Korntal-Münchingen, Germany) in them to Geno grind later. The samples were measured on a scale and immediately frozen in liquid nitrogen for further analysis.

### 4.3. Verticillium dahliae Growth Condition and Co-Cultivation

*V. dahliae* (FSU-343) was collected from the Jena Microbial Resource Centre, Jena, Germany. A plug of *V. dahliae* was grown on the center of a PDA plate at 23 °C in the dark for 1–2 weeks to obtain a fungal lawn. To obtain a high spore count a plug of mycelia at the white color stage from a previously grown fungal lawn was added into a liquid KM medium [37]. The liquid KM media with an added inoculum was kept on a shaker at 110 rpm in the dark at room temperature for 4–5 days. The fungal culture was filtered through 3–4 layers of sterile nylon membranes. The collected spores were washed with sterile water, centrifuged, and the supernatant was thrown. The procedure was repeated 3 times. The Haemocytometer was used to determine the spore count and was adjusted to 5 × 10^6^ per mL. Two days before the co-cultivation experiment 100 µL of collected *V. dahliae* spores (in water) were spread onto 6 nylon membrane stripes (1 cm × 4 cm size each) on a PDA plate. The same setup was conducted separately with water as a control. Both *V. dahliae* stripe plates and a control group’s plates were incubated at room temperature in the dark. Once the plants reached 10 days of age, they were ready to be infected with fungus. The nylon stripes incubated with *V. dahliae* spore suspension were shifted onto new separate PNM media plates (4 stripes per Petri plate). The same procedure was conducted for the control group. The 10-day-old seedlings were transferred to PNM plate which was previously laid with nylon membranes. The single seedling was laid horizontally over one rectangular (1 cm × 4 cm) stripe. While laying the seedling, roots were kept over the stripe and the shoot was kept a few mm distant from the stripe on a bare PNM medium. Hence, the fungus did not directly touch the leaves [38]. The plants of the control group were laid over nylon stripes which were treated with sterile water. The Petri dishes were sealed with 3M^TM^ Micropore tape (3M Germany GmbH, Neuss, Germany). The fungus and control group of Petri dishes were incubated at 22–24 °C with a 16 h light/8 h dark photoperiod for 7 days at 80 µmol m^−2^ s^−1^ light intensity. After that, the roots and shoots were cut, washed with sterile water, and wiped with tissue paper. The weight of the shoot and root was measured on a scale and collected separately in the sterile Sarstedt Screw Cap Microtubes (Nümbrecht, Germany) with two sterile metal beads (4.5 mm diameter, Askubal, Korntal-Münchingen, Germany) in them to Geno grind later. The samples were frozen immediately in liquid nitrogen for further analysis.

### 4.4. Animal Material and Feeding Assay

Eggs of the generalist herbivore *Spodoptera littoralis* Boisd (Lepidoptera, Noctuidae) were obtained from Syngenta Crop Protection AG (Stein, Switzerland). Larvae were hatched and reared on an artificial diet with a 10 h light/14 h dark photoperiod at 23 to 25 °C [39]. First-instar larvae were used for the feeding assay experiment that was conducted as described [29]. Different sets of 30 larvae per bunch were weighed, each bunch weighing about 60 mg in total. Three larvae per plant were placed for feeding. Each whole plant was covered with a special plastic bag composed of a mesh-like structure for breathing purposes. The whole experiment was carried out inside a short-day growth chamber. Seven days later the larvae were collected and weighed after feeding on *A. thaliana* wild type, TIRP KO, and TIRP OE lines.

### 4.5. Spodoptera littoralis Egg Extract Treatment

Eggs of *S. littoralis* were crushed in an Eppendorf tube with a pestle. The tubes were centrifuged at 15,000× *g* for 3 min. After centrifugation, the supernatant was collected and transferred into small PCR tubes as aliquots [17]. Approximately 100 eggs weighed around 6.5 to 7 mg to obtain 2 µL of extract as a single aliquot. The egg extract was collected and stored at −20 °C. The five-week-old plants of *A. thaliana* wild type, TIRP KO, and TIRP OE were used for the experiment. The 2 µL of egg extract was taken by micropipette and applied on the bottom part of the leaf to mimic the eggs laying of larvae on the leaf. A single leaf per plant was selected and left in a short-day growth chamber. After 48 h the dried layer of egg extract was removed by a brush and a leaf was collected in a Sarstedt Screw Cap Microtubes (Nümbrecht, Germany) tube and frozen immediately in liquid nitrogen for further analysis.

### 4.6. Phytohormone Analysis

The phytohormones were extracted from the leaf and root part of the plants. In Sarstedt Screw Cap Microtubes (Nümbrecht, Germany) two sterile metal beads (4.5 mm diameter, Askubal, Korntal-Münchingen, Germany) were added and around 50 to 100 mg of samples were collected. The samples were ground in Geno/Grinder 2010 SPEX CertiPrep^TM^ (Fisher Scientific GmbH, Schwerte, Germany) for 30 s at 1000 rpm. The ground samples were mixed with 1 mL of methanol containing 40 ng mL^−1^ of D4-SA (Santa Cruz Biotechnology, Dallas, TX, USA), D6-JA and 8 ng mL^−1^ of D6-JA-Ile (both HPC Standards GmbH, Cunnersdorf, Germany) [40]. The samples were kept in a shaker at 4 °C for 30 min. After that, the tubes were centrifuged for 13,000× *g* at 4 °C for 20 min. The supernatant was transferred to the new 2 mL Eppendorf tube on ice. To the pellet in the previous tube 500 µL of methanol was added. Once again, the vortex and centrifugation were repeated as mentioned in the previous step. The supernatant was collected in the same previous 2 mL Eppendorf tube. The combined supernatant was kept inside the vacuum concentrator (Eppendorf Concentrator Plus, Wesseling-Berzdorf, Germany) for drying on the V-AL setting at 30 °C for 2 h. Later 500 µL of methanol was added to the dried residues and vortexed and centrifuged for 16,000 rpm at 4 °C for 5 min. The 400 µL of supernatant was transferred into HPLC vials. Phytohormone analysis was performed by LC-MS/MS as described in Heyer et al. [41] on an Agilent 1260 series HPLC system (Agilent Technologies, Santa Clara, CA, USA) with the modification that a tandem mass spectrometer QTRAP 6500 (SCIEX, Darmstadt, Germany) was used. Chromatographic separation was achieved on a Zorbax Eclipse XDB-C18 column (50 × 4.6 mm, 1.8 µm, Agilent Technologies, Santa Clara, CA, USA). Waters containing 0.05% formic acid and acetonitrile were employed as mobile phases A and B, respectively. The elution profile was as follows: 0–0.5 min, 10% B; 0.5–4.0 min, 10–90% B; 4.0–4.02 min, 90–100% B; 4.02–4.5 min, 100% B, and 4.51–7.0 min, 10% B. Flow rate was kept at 1.1 mL min^−1^ and column temperature was maintained at 25 °C. The mass spectrometer was equipped with a Turbo spray ion source operated in negative ionization mode. The ion spray voltage was maintained at −4500 eV. The turbo gas temperature was set at 650 °C. Nebulizing gas was set at 60 psi, curtain gas at 40 psi, heating gas at 60 psi, and collision gas was set to “medium”. The mass spectrometer was operated in multiple reaction monitoring (MRM) mode; details of the instrument parameters and response factors for quantification can be found in Table S2. Since we observed that both the D6-labeled JA and D6-labeled JA-Ile standards (HPC Standards GmbH, Cunnersdorf, Germany) contained 40% of the corresponding D5-labeled compounds, the sum of the peak areas of D5- and D6-compound was used for quantification.

### 4.7. Gene Expression Analysis

The 50 to 100 mg sample was collected in Sarstedt Screw Cap Microtubes (Nümbrecht, Germany) and ground in a Geno/Grinder 2010 SPEX CertiPrep^TM^ (Fisher Scientific GmbH, Schwerte, Germany) for 30 s at 1000 rpm. The RNase-free microtubes were pre-loaded with two sterile grinding metal beads (4.5 mm diameter, Askubal, Korntal-Münchingen, Germany) before grinding. The RNA was extracted by using Invitrogen TRIzol by Thermo Fisher Scientific (Waltham, MA, USA) and followed the protocol provided in the kit. To make the isolated RNA free of genomic DNA contamination it was treated with DNA digestion. For the digestion of DNA, the Invitrogen TURBO DNase kit (Thermo Fisher Scientific, Carlsbad, CA, USA) was used following the manufacturer’s protocol. The RevertAid First Strand cDNA Synthesis kit by Thermo Fisher Scientific was used for making complementary DNA (cDNA) from the RNA. The protocol provided by the kit was used for synthesizing the cDNA. The primers specific to TIRP can be found in Table S1. The quantitative real-time PCR analysis was performed on the CFX96 Real-Time PCR detection system, with CFX Maestro Software version 1.1 (Bio Rad, Hercules, CA, USA). The Brilliant II SYBR green master mix (Agilent, Santa Clara, CA, USA) was used. Each biological sample was analyzed in 3 technical replicates. The qPCR program was initiated at 95 °C for 3 min; the 44 cycles consisted of 95 °C for 30 s, 60 °C for 30 s, and 72 °C for 30 s and the melting curve was determined between 65 °C and 95 °C. The relative target gene expression was normalized to the reference gene Actin2. The primers can be found in Table S1. The normalized fold expression was calculated according to ΔΔCT [42].

### 4.8. Statistical Analyses

All statistical analyses and graph composition were performed by using GraphPad Prism version 9.4.1 software. Statistical differences were shown by Student *t*-test and two-way ANOVA (factors: treatment and *Arabidopsis* lines) followed by Tukey’s post hoc test. The biological replicates have been mentioned in the legends of respected figures.

## 5. Conclusions

The role of TIR-X (TX) proteins in plants is still not known although some reports suggest their involvement in plant immunity. Our study was designed from the outset to investigate the role of a defined TIR-X in plants under attack, namely that of TIRP, which has never been done before. Based on the literature [8,9], it was conceivable that plant TIR-X proteins might have a role during any kind of attack. We challenged the hypothesis that TIRP is involved in phytohormone-mediated defense regulation when the plant is attacked by either a necrotrophic fungus, a hemibiotrophic fungus, or herbivorous insects. Based on the analysis of salicylic acid (SA) and jasmonates phytohormones in WT, TIRP knock-out (KO), and TIRP overexpressing (OE) *Arabidopsis thaliana* plants this hypothesis could not be confirmed. Nevertheless, without any treatment the TIRP overexpressing plants have a significantly higher SA level compared to the WT (Col-0) and TIRP KO lines. The finding that in WT plants upon *A. brassicicola* and *V. dahliae* infection the TIRP gene expression was induced suggests that it is influenced by pathogen attacks. Since there was nothing known about the herbivory effect on *Arabidopsis* TX, we studied this as well. Here, the higher content of SA in the TIRP OE line might explain the finding that *S. littoralis* larvae gained more weight when feeding on the TIRP OE line compared with WT and TIRP KO lines, having the SA and jasmonate antagonism in mind. Together with the few other studies on TIR-X genes showing that overexpressing lines exhibit HR and resistance against some pathogens, we can conclude that some TIR-X-proteins might be involved in plant immunity, at least to some extent. Because for TIRP this is phytohormone independent, the molecular mechanisms are still unknown and we need more work for a deeper understanding.

## Data Availability

Data is contained within the article and Supplementary Materials.

## Supplementary Materials

The supporting information can be downloaded at https://www.mdpi.com/article/10.3390/ijms26062764/s1.

## Abbreviations

The following abbreviations are used in this manuscript:

TIRs	Toll/Interleukin-1 receptors
PTI	Pattern-triggered immunity
ETI	Effector-triggered immunity
PRRs	Pattern recognition receptors
WT	Wild type
KO	Knock-out
OE	Overexpressed
NBS	Nucleotide-binding site
R	Resistance protein
LRR	Leucine Rich Repeat
CC	Coiled coil
HR	Hypersensitivity response
CFU	Colony forming unit
PDA	Potato dextrose agar
PDB	Potato dextrose broth
MS	Murashige and Skoog
VDA	*Verticillium dahliae*
SA	Salicylic acid
JA	Jasmonic acid
JA Ile	Jasmonoyl isoleucine
LC-MS/MS	Liquid chromatography-mass spectrometry/mass spectrometry
SNE1	Suppressor of necrosis-1
NLPs	Nep1-like proteins
NPR1	Non-expresser of PR genes
EDS1	Enhanced disease susceptibility 1
PAD4	Phytoalexin-deficient 4
SAG101	Senescence-associated gene 101
PEK	Pieris egg-killing
