# Supplementary material for: Exploring a Role for the Arabidopsis TIR-X Gene (TIRP) in the Defense Against Pathogenic Fungi or Insect Herbivory Attack
Source: Int J Mol Sci. 2025 Mar 19;26(6):2764. doi: 10.3390/ijms26062764 (PMC11943168; doi:10.3390/ijms26062764)
Supplement: Supplementary file 1 [file ijms-26-02764-s001.zip › ijms-3446278-supplementary.pdf]

Supplementary Materials

Exploring a Role for the Arabidopsis TIR-X Gene (TIRP) in the Defense Against Pathogenic Fungi or Insect Herbivory Attack

Shraddha Neufeld, Michael Reichelt, Sandra S. Scholz, Przemysław Wojtaszek and Axel Mithöfer

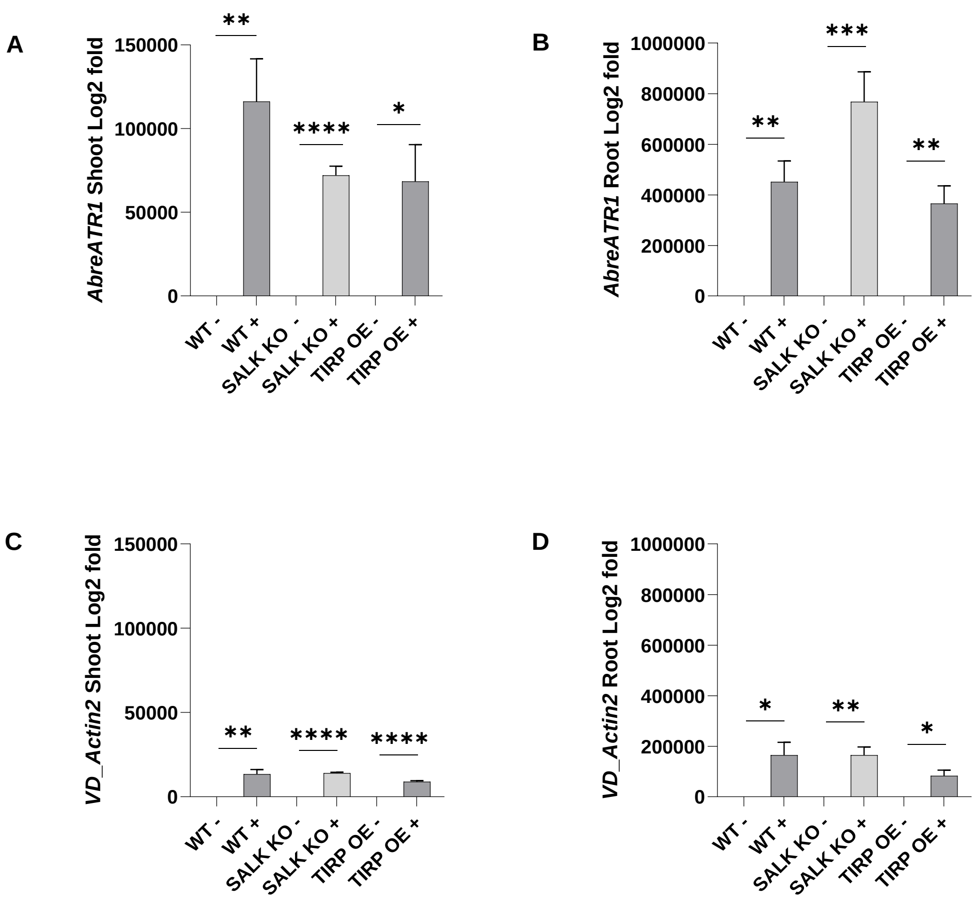

Figure S1: Fungal gene expression analysis to prove successful infections and colonization in *Arabidopsis* WT, TIRP KO, or TIRP OE lines. (A, B) *AbreATR1* gene expression level for *Alternaria brassicae*-infection in shoots and roots. A single leaf per seedling (10-day-old) was infected with necrotrophic *A. brassicae* spores. (C, D) *VD Actin2* gene expression levels for *Verticillium dahliae* infection in shoots and roots. Roots (10-day-old) were infected with hemibiotrophic *V. dahliae*. After 7 days of infection, shoots and roots were separated and qPCR analysis was done. Shown are the means ±SE. Statistical differences were calculated using two-way ANOVA and considered significant when $P \leq 0.05$ (Tukey). (*) represents significant differences, (**) represents $P \leq 0.01$, (***) represents $P \leq 0.00$, (****) represents $P \leq 0.0001$. *A. brassicae* treatment WT- n = 96, WT+ n = 80, TIRP KO- n = 84, TIRP KO+ n = 92, TIRP OE- n = 88, TIRP OE+ n = 84. *V. dahliae* treatment WT- n = 112, WT+ n = 104, TIRP KO- n = 112, TIRP KO+ n = 108, TIRP OE- n = 108, TIRP OE+ n = 112 from four independent repetitions. The label (+) means with fungal infection; (-) means without fungal infection.

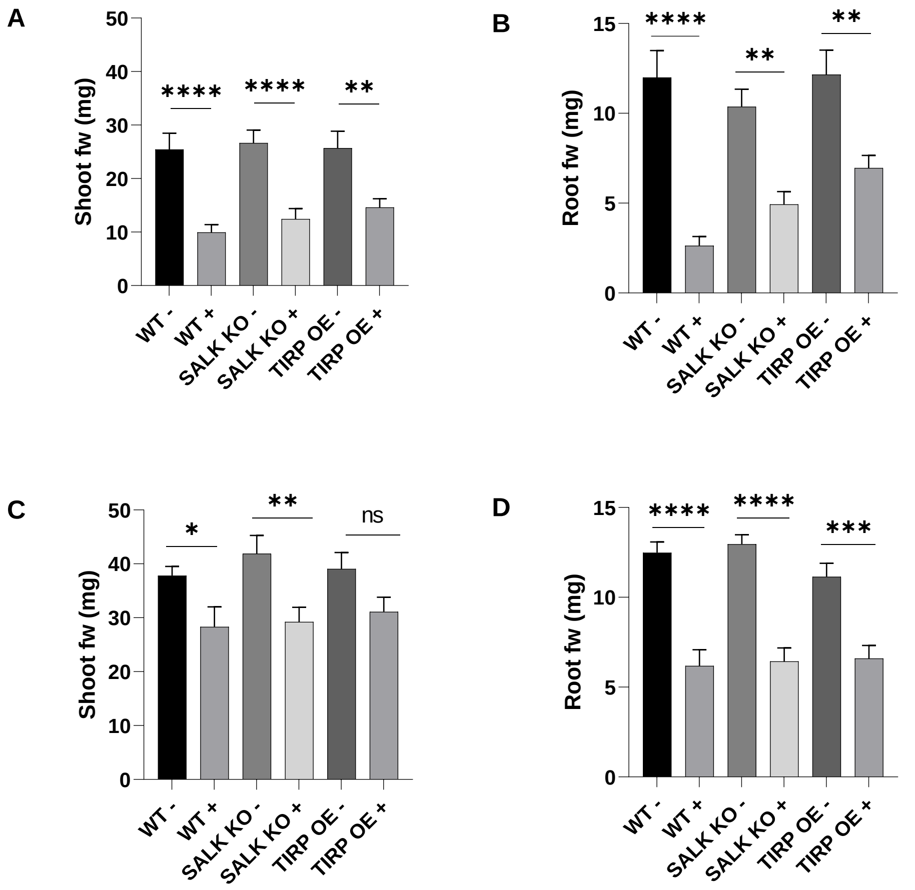

Figure S2: Weight changes in shoots and roots of fungal-infected *Arabidopsis* WT, TIRP KO or TIRP OE lines. (A, B) A single leaf per seedling (10-day-old) was infected with necrotrophic *A. brassicae* spores. (C, D) Roots (10-day-old) were infected with hemibiotrophic *V. dahlia* (VDA). After 7 days of infection, shoots and roots were separated and weighed. Shown are the means ±SE- Statistical differences were calculated using two-way ANOVA and considered significant when $P \leq 0.05$ (Tukey). The ns represents non-significant, $P > 0.05$, (*) represents $P \leq 0.05$, (**) represents $P \leq 0.01$, (***) represents $P \leq 0.001$, (****) represents $P \leq 0.0001$. *A. brassicae* treatment WT- n = 96, WT+ n=80, TIRP KO- n = 84, TIRP KO+ n = 92, TIRP OE- n = 88, TIRP OE+ n = 84. *V. dahliae* treatment WT- n = 112, WT+ n = 104, TIRP KO- n = 112, TIRP KO+ n = 108, TIRP OE- n = 108, TIRP OE+ n = 112 from four independent repetitions. The label (+) means with fungal infection; (-) means without fungal infection.

Table S1: Primers used for RTqPCR.

Primer name	Sequence 5´ to 3´	Target
TIRP forward	CGAGGAATCGAGAGTCGCAT	AT5G44900
TIRP Reverse	CGCGATGCGTTAACTTCAGG	AT5G44900
TIRP KO forward	TTTTGGTGTTTTCGTGGAGAG	SALK 014983
TIRP KO reverse	TTAACCTCCTCGACGATTGTG	SALK 014983
Actin2 forward	CAATCTCATCTTCTTCCGCTCTTT	AT3G18780
Actin2 reverse	CAAATCCAGCCTTCACCATACC	AT3G18780
VD_Actin2 forward	CGTTCAGGTCATCACGCA C	VDAG_07506
VD_Actin2 reverse	TATTCTTGGTACTCCGCCTTG	VDAG_07506
AbreATR1 forward	ACCCGCATTCCTCGCCAAA	AY246696.1
AbreATR1 reverse	AAGTCAAGGATTGTGTCGAGCTT	AY246696.1

Table S2. Details of analysis of phytohormones by LC-MS/MS in negative ionisation mode

Q1	Q3	RT (min)	Compound	Internal std	RF	DP	CE
**136.93**	93.00	3.3	SA	D4-SA	1.0	-20	-24
**209.07**	59.00	3.6	JA	D6-JA	1.0	-20	-24
**322.19**	130.10	3.9	JA-Ile	D6-JA-Ile	1.0	-50	-30
**140.93**	97.00	3.3	D4-SA			-20	-24
**215.00**	59.00	3.6	D6-JA			-20	-24
**214.00**	59.00	3.6	D5-JA			-20	-24
**328.19**	130.10	3.9	D6-JA-Ile			-50	-30
**327.19**	130.10	3.9	D5-JA-Ile			-50	-30
